# Health practitioners’ state of knowledge and challenges to effective management of hypertension at primary level

**DOI:** 10.5830/CVJA-2010-066

**Published:** 2011-08

**Authors:** A Parker, B Nagar, G Thomas, NBA Ntusi, M Badri

**Affiliations:** Department of Medicine, Groote Schuur Hospital and the University of Cape Town, South Africa; Department of Medicine, Groote Schuur Hospital and the University of Cape Town, South Africa; Department of Medicine, Groote Schuur Hospital and the University of Cape Town, South Africa; Department of Medicine, Groote Schuur Hospital and the University of Cape Town, South Africa; Clinical Research Support Unit, Department of Medicine, Groote Schuur Hospital and the University of Cape Town, South Africa

**Keywords:** hypertension, primary healthcare, South Africa, challenges to healthcare

## Abstract

**Background:**

Patient- and physician-related factors impact on the management and control of hypertension.

**Objectives:**

To systematically examine: (1) South African primary care doctors’ state of knowledge on the management of hypertension; (2) primary health practitioners’ knowledge on the South African hypertension guidelines; (3) current approaches to management of hypertensive patients; and (4) challenges to effective management of hypertension at primary level.

**Methods:**

A cross-sectional, observational study using a semi-structured questionnaire was carried out in two large community health centres (CHCs) in the Cape Town metropole. All 16 doctors employed at both CHCs were voluntarily enrolled, seven (43.7%) of whom were female, with 14 (87.5%) younger than 40 years of age. The majority (81.2%) of the doctors surveyed had been practicing for less than 10 years.

**Results:**

Ten (62.5%) of the doctors surveyed aimed to treat hypertension to target, and recommendations on lifestyle modifications were reportedly poorly done. While 11 (68.8%) of the doctors were aware of the South African hypertension guidelines, were (81.8%) of them were not conversant with the contents thereof. Doctors estimated that only 35% of their patients are treated to target. Poor patient adherence to prescribed treatment, language difficulty, heavy patient load, medical staff shortages, and patient loss to follow up were identified by the doctors as significant impediments to the effective management of hypertension at the primary level of care.

**Conclusion:**

Primary healthcare practitioners’ knowledge regarding hypertension and the South African hypertension guidelines is poor. Management of hypertension by these doctors is sub-optimal. There are significant challenges to effective management of hypertension at this level of care.

## Abstract

Hypertension is common^1^ and it increases cardiovascular morbidity and mortality.^2^ In 2001, 54% of all strokes and 47% of all ischaemic heart disease were due to hypertension, with 80% of this blood pressure-attributable disease burden occurring in low-and middle-income countries. More than half occurred in people of working age (45 to 69 years of age).^1^ A recent Canadian study demonstrated that a simplified hypertension treatment algorithm administered to 45 general practices resulted in a significantly higher proportion of patients achieving their target blood pressure.^3^

Factors that impact on doctors’ ability to provide good management of hypertension have previously been investigated.^4^ Both patient- and physician-related factors have significant impact on blood pressure control in hypertensive patients.^5^ The South African Hypertension Society published guidelines, designed with expert consensus, to guide the management of hypertension in South Africa, to minimise the gap between the public and private sectors of healthcare, and to improve care of individuals with hypertension.^6^ Yet, the publication of these guidelines does not seem to have significantly impacted on the management practice of many doctors who treat hypertension.

There have been few studies in South Africa that have systemically examined doctors’ impressions of the phenomena that impede effective care of their hypertensive patients. Hence, the objectives of the study were to systematically examine: (1) South African primary care doctors’ state of knowledge on the management of hypertension; (2) primary health practitioners’ knowledge on the South African hypertension guidelines; (3) current approaches to management of hypertensive patients; and (4) challenges to effective management of hypertension at primary level.

## Methods

Doctors working at two large community health centres (CHCs) with a predominantly black patient population in the Cape Town metropole were selected to participate in the study. The study was designed to be an observational study, with the data-collection tool in the form of a semi-structured questionnaire. The investigators conducted all interviews and assessments in English over a four-week period in 2008. Informed consent for inclusion in the study was obtained from all the doctors.

The data in this study were collected as part of a project for a fifth-year primary health block for the medical students enrolled at the University of Cape Town Faculty of Health Sciences. Permission for analysis of the data for the purpose of publication was obtained from the University of Cape Town Health Sciences Faculty Research Ethics Committee. Relevant statistical analysis using Microsoft Excel, SPSS and STATISTICA was performed. Normally distributed data were analysed using the Student’s *t*-test, and non-parametric data were analysed using either the Fisher’s exact test or Pearson chi-square test. All *p*-values were two-sided, and *p* < 0.05 were taken to indicate statistical significance.

## Results

There were 16 doctors employed at the two CHCs, all of whom were voluntarily enrolled into this study. Seven (43.7%) of the doctors were female, 14 (87.5%) were aged between 26 and 40 years, and 81.25% of the doctors surveyed had been practicing medicine for less than 10 years (Table 1).

**Table 1.** Demographic Details Of Doctors Surveyed

*Demographic details*	*Doctors surveyed (n = 16)*
Gender	
male (%)	9 (56.3)
female (%)	7 (43.7)
Age (years)	
< 25 (%)	1 (6.25)
26–30 (%)	7 (43.75)
31–40 (%)	7 (43.75)
over 60 (%)	1 (6.25)
Duration of practice (years)	
0–5 (%)	8 (50.0)
6–10 (%)	5 (31.25)
11–15 (%)	1 (6.25)
16–20 (%)	1 (6.25)
> 35 (%)	1 (6.25)

## Management of hypertension in general

All the doctors (100%) felt that the management of hypertension was a significant part of their daily practice. Ten (62.5%) stated that they attempted to treat hypertension to target. All doctors thought that lifestyle modification was an important adjunct to the treatment of hypertension, yet recommendations on lifestyle modifications to patients were reportedly poorly done by all the doctors. Only 50% of doctors indicated that they even mentioned lifestyle modifications to some of their patients (Table 2).

**Table 2.** Knowledge About Hypertension And Preference For Management Of Hypertension Among Doctors Surveyed

Proportion who felt that hypertension is an important aspect of their daily practice (%)	16 (100.0)
Proportion who attempt to treat hypertension to target (%)	10 (62.5)
Proportion who feel that lifestyle modification is an important aspect of management of hypertension (%)	16 (100.0)
Proportion who recommend lifestyle modification to their patients (%)	8 (50.0)
Proportion who are aware of the existence of the hypertension guidelines (%)	11 (68.8)
Proportion who are conversant with the contents of the hypertension guidelines (%)	2 (12.5)
Choice of drugs to treat hypertension, in the absence of compelling indications	11 (68.8)
First line: hydrochlorothiazide	11 (68.8)
Second line: enalapril	10 (62.5)
Third line: amlodipine	5 (31.25)
Fourth line: beta-blocker, hydralazine or other agent	

## Awareness of the South African hypertension guidelines

**Fig. 1.**
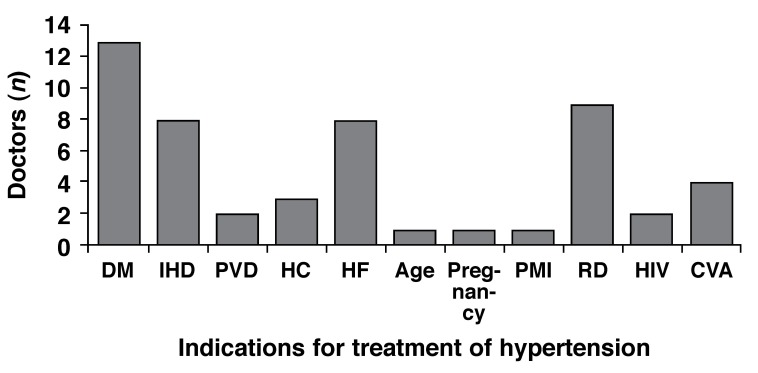
Compelling indications for the treatment of hypertension as given by the doctors surveyed. DM = diabetes mellitus, IHD = ischaemic heart disease; PVD = peripheral vascular disease, HC = hypercholesterolaemia, HF = heart failure, PMI = previous myocardial infarction, RD = renal disease, HIV = human immunodeficiency virus infection, CVA = cerebrovascular accident.

Eleven (68.8%) of the doctors were aware of the recent South African hypertension guidelines. Of these 11, nine (81.8%) stated that they were not conversant with the content of the guidelines (this was reported in spite of the guidelines being prominently displayed in the corridors and some of the consulting rooms of both CHCs). Knowledge of the compelling indications for treatment of hypertension, as stated in the hypertension guidelines, was poor, with the majority of doctors not knowing what these conditions are (Fig. 1).

## Estimation of effective blood pressure control

Overall, the doctors estimated that 35% (range 5–60%) of their hypertensive patients were controlled on the antihypertensive treatment prescribed.

## Preferred management approach to hypertension

Doctors were asked to list their preferred treatment for hypertension. Eleven (68.8%) of the doctors stated that hydrochlorothiazide was their preferred first-line antihypertensive agent, in the absence of compelling indications; with 11 (68.8%) indicating that enalapril was their preferred second-line agent. Ten (62.5) selected amlodipine as a preferred third-line agent. Four (25%) of the participants in the study indicated that b-blockers were their preferred fourth-line antihypertensive, with various other drugs listed as fourth-line options, in the absence of compelling indications (Table 2).

**Fig. 2.**
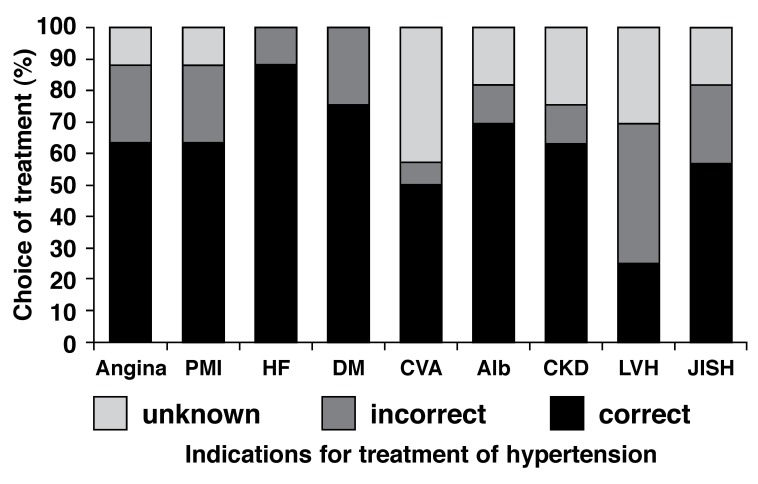
Choice of preferred antihypertensive agent, when these compelling indications are present. Angina = ischaemic heart disease or angina pectoris, PMI = previous myocardial infarct, HF = heart failure, DM = diabetes mellitus, CVA = prior cerebrovascular accident, PVD = peripheral vascular disease, Alb = albuminuria, CKD = chronic kidney disease, LVH = left ventricular hypertrophy, and JISH = just isolated systolic hypertension.

Doctors were also asked to choose from a list of four drugs (angiotensin converting enzyme inhibitor or angiotensin receptor blocker, calcium channel blocker, β-blocker or diuretic) and indicate their preferred choice of antihypertensive agent, when the following compelling indications are present: ischaemic heart disease or angina, heart failure, diabetes mellitus, prior cerebrovascular accident, peripheral vascular disease, albuminuria, chronic kidney disease, left ventricular hypertrophy, and isolated systolic hypertension. Knowledge of the compelling indications for treatment of hypertension was poor, with the majority (62.5%) of doctors not knowing what the appropriate agent of choice for these conditions should be (Fig. 2).

## Factors influencing optimal management of hypertension

The main challenges to optimal treatment of hypertension in their settings, as reported by doctors in this study, included: (1) poor patient adherence to prescribed treatment (75%); (2) language difficulty (50%); (3) overwhelmingly heavy patient load (50%); (4) significant medical staff shortages (50%); and (4) patient loss to follow up (44%). Other factors that were mentioned included conditions of adverse poverty under which many patients live; poor patient literacy; intermittent shortage or lack of drugs; lack of functional equipment (including sphygmomanometers); and other systematic factors (Table 3).

**Table 3.** Proportion Of Doctors Who Identified Factors Influencing Optimal Management Of Hypertension

Poor patient treatment adherence	12 (75.0)
Language difficulty	8 (50.0)
Overwhelming patient load	8 (50.0)
Severe staff shortages	8 (50.0)
Patient loss to follow up	7 (43.75)
Poverty	4 (25.0)
Poor patient literacy	3 (18.75)
Lack of drugs	2 (12.5)
Lack of functional equipment	2 (12.5)
Systematic factors like financial constraints on tests	3 (18.75)

## Discussion

The findings of this study suggest that the knowledge of South African primary healthcare practitioners regarding hypertension and its management is sub-optimal. Knowledge on the South African hypertension guidelines is poor. Doctors estimated that about two-thirds of their hypertensive patients have poor blood pressure control, and yet treatment for these patients is not routinely titrated upwards. Moreover, the doctors who participated in this study identified significant challenges to their effective management of hypertension.

Various stakeholders in the healthcare sector will have to work together to address these challenges if we are to improve care of hypertensive patients in this country. Furthermore, ongoing education of doctors is crucial in order to increase knowledge on hypertension and awareness of the management guidelines and to encourage them to overcome physician inertia. On a positive note, it is interesting to observe that β-blockers were not considered as first-line treatment of hypertension, in the absence of compelling indications.

Despite hypertension being identified as an important aspect of the practice of doctors at the primary healthcare level, many doctors do not focus on lifestyle modifications. The reason for this phenomenon, as reported by the doctors surveyed, is the lack of adequate time at each consultation for explanation to patients about necessary lifestyle changes to complement their drug treatments.

The sixth report of the Joint National Committee on prevention, detection, evaluation and treatment of high blood pressure included evidence-based lifestyle modifications that have been shown to lower blood pressure in normotensive and hypertensive patients.^7^ These important lifestyle modifications include weight loss of 3 to 9%, moderation in alcohol use, smoking cessation, increased physical activity, reduced dietary salt intake, reduced intake of saturated fats and cholesterol, and adequate dietary intake of potassium, calcium and magnesium. Each of these lifestyle changes have the effect of lowering systolic blood pressure by 3 to 11 mmHg and the diastolic blood pressure by 2.5 to 5.5 mmHg.^8^-^12^

Therefore, as drugs fail to adequately control blood pressure in the majority of patients, these lifestyle changes are important adjuncts in the initial and comprehensive management of patients with an elevated blood pressure. These lifestyle alterations should be enforced at the primary level of care, rather than waiting for patients to develop complications and to receive these messages from secondary and tertiary hospitals, when they may be too late.

A disturbing finding was that a significant proportion (37.5%) of doctors surveyed did not aim to treat patients to target. Despondency on the part of overwhelmed healthcare practitioners, including doctors in the primary level of care within the public sector in South Africa, in the management of hypertension has previously been documented by several authors.^7^,^13^,^14^ The net effect of these despondent attitudes towards patient care is further compromising an already crumbling management paradigm.

Almost 90% of doctors in this study were below the age of 40 years, with most having practiced as doctors for 10 years or less. This observation reflects the state of healthcare in South Africa, where fairly junior doctors are entrusted with enormous clinical responsibility. While these young doctors grow quickly in their trade and benefit from having to assume responsibility for the management of their patients, patient care is not always optimal. Availability of increased numbers of senior doctors and specialists who provide outreach may go a long way in ameliorating some of these challenges.

Another disconcerting finding in this study was the observation that most doctors were unable to name the compelling indications for hypertension treatment or failed to list the correct treatment when given a list of these conditions. This demonstrates that there is a need for ongoing education of doctors about management of conditions with important public health implications, such as hypertension. The publication of guidelines is an important way of developing minimum standards for coherence and uniformity in treatment of clinical entities. However, as demonstrated in this study, the prominent display of guidelines in hospital corridors and consulting rooms does not equate to physician knowledge.

An interesting question that arises from this study is: how can primary health practitioners who treat one of the most common conditions in their practice, hypertension, be unfamiliar with or ignorant of the latest guidelines? There is a perceived, and perhaps real disconnect between experts (who are mostly academics) who write guidelines, and primary healthcare doctors (who are expected to implement guidelines and improve health of the population), with limited opportunities for engagement between the two groups.

After publication of guidelines, dissemination of the message to the relevant doctors is of paramount importance. Publication in journals and posters is clearly not sufficient. Multi-pronged implementation and education programmes need to be developed. Others have demonstrated that education alone is not enough to change physician behaviour, and that the process of change is more related to attitude.^15^,^16^

In other parts of the world, where there is also poor implementation of hypertension guidelines by primary health doctors, authors have demonstrated that hypertension clinical guidelines are often regarded as optional rather than standards, and many doctors feel that the recommendations are not suitable for their patients.^17^ While it is not clear if such attitudes are also operational in our small sample of doctors, it is clear that multi-dimensional risk stratification and intervention is too time-consuming for doctors who are already overwhelmed by the workload of patients.

An important aspect of this study was the examination of factors that impact on the ability of public-sector primary healthcare doctors to effectively treat hypertension. In the main, these challenges were related to reported poor adherence to treatment by patients, communication difficulties due to doctors not speaking the language of the patients, and heavy patient load in the context of significant shortage of both nurses and doctors. A further challenge elaborated on by the doctors is the fact that many patients regularly move between the Eastern Cape and Western Cape provinces, with the consequence of regular patient loss to follow up. High levels of poverty and illiteracy were also mentioned as factors that hamper effective care of patients. Drug shortages and lack of functional equipment are problems that also emerge, from time to time, to affect care of patients with hypertension and other chronic co-morbidities. Systematic factors, including financial restrictions on investigation of patients at the primary level of care were also mentioned by some of the doctors.

Interestingly, in a different study by Steyn and colleagues, conducted in a random sample of 18 CHCs in the Cape Town area, the authors similarly found that staff shortages, complexity of cases previously managed in tertiary hospitals and lack of financial resources for special investigations were reported by healthcare workers as significant impediments to their care of patients with chronic conditions.^18^

## Conclusion

This study demonstrates that the majority of doctors treating hypertension in the primary health clinics are fairly junior, and significant gaps exist in their knowledge regarding management of hypertension. Awareness of the South African hypertension guidelines should be improved. Furthermore, an urgent, concerted, multi-sectorial effort to address the challenges to effective care of hypertensive patients at a primary level of care is needed.
